# Good outcomes after the delayed removal of an epidural hematoma: A case report

**DOI:** 10.1097/MD.0000000000010341

**Published:** 2018-04-06

**Authors:** Yong-Xing Yao, Ming-Xia Li, Lian-Juan Sun

**Affiliations:** Department of Anesthesia, First Affiliated Hospital, Zhejiang University School of Medicine, Hangzhou, China.

**Keywords:** aortic aneurysm, epidural hematoma, spinal anesthesia

## Abstract

**Rationale:**

Epidural hematomas can develop following intrathecal puncture, spinal vascular malformation, or spontaneous hemorrhage. Prompt recognition of symptoms and referral to neurosurgical services are crucial for recovery. We report a rare case of delayed removal of an epidural hematoma that resulted in good patient outcomes.

**Patient concerns:**

A 69-year-old woman weighing 58 kg presented with back pain that had persisted for 12 years. Her medical history was notable for hypertension. Cervical computed tomography angiography indicated a giant abdominal aortic aneurysm measuring 11.58 × 17.34 × 13.00 cm in her abdominal cavity. Stent graft implantation was scheduled by the interventional therapy group to treat her abdominal aortic aneurysm under single-dose spinal anesthesia. Postoperatively, the patient complained of numbness and swelling of her lower extremities, with progressive paralysis developing in both legs accompanied with sensory disturbances.

**Diagnoses:**

Delayed magnetic resonance imaging detected an epidural hematoma.

**Interventions:**

Eighty hours after the onset of sensorimotor symptoms, lumbar decompression and removal of the hematoma were performed via laminectomy under general anesthesia.

**Outcomes:**

The patient was discharged 2 months after the operation with a 3/5 score on the Muscle Strength Grading System.

**Lessons:**

Laminectomy may be effective in achieving a good prognosis despite a considerable delay in diagnosis.

## 1 Introduction

Epidural hematoma may result from an intrathecal puncture (either needle or catheter) to the blood vessels, congenital vascular malformation, or spontaneous hemorrhage.^[1,2]^ Clinical symptoms vary in accordance with the degree of spinal compression. In patients using anticoagulant drugs, intrathecal anesthesia is a common cause of epidural hematoma in the clinic.^[3]^ Once diagnosed, prompt intervention with laminectomy within 8 h of onset is crucial to recovery.^[4]^ Failure to appropriately manage an epidural hematoma often results in permanent neurological injury. We present a case in which good outcomes were achieved despite delayed removal of an epidural hematoma in a patient who underwent stent graft implantation for an abdominal aortic aneurysm.

## 2 Case presentation

**Figure 1:**
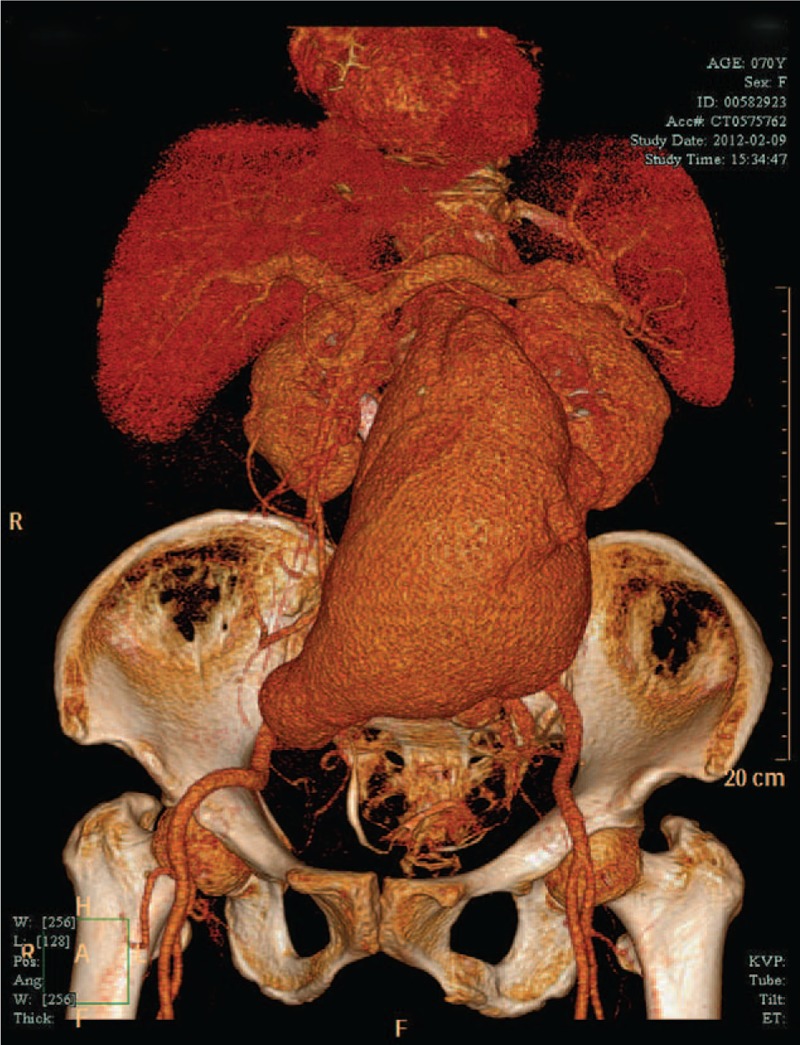
Preoperative computed tomography angiography indicated a large abdominal aortic aneurysm.

A 69-year-old woman weighing 58 kg presented with back pain that had persisted for 12 years. Her medical history was remarkable for hypertension, which was treated with compound hypotensive tablets. Cervical computed tomography angiography showed an abdominal aortic aneurysm measuring 11.58 × 17.34 × 13.00 cm in her abdominal cavity (Fig. 1). Routine preoperative laboratory tests revealed that she had normal hemoglobin concentration (126 g/L); normal blood coagulation function, with a prothrombin time of 11.2 s; and a normal platelet count (163 × 10^9^/L), but had a compromised pulmonary function, with an elevated PaCO_2_ (52.6 mm Hg). Anticoagulants were not prescribed perioperatively for this patient.

**Figure 2:**
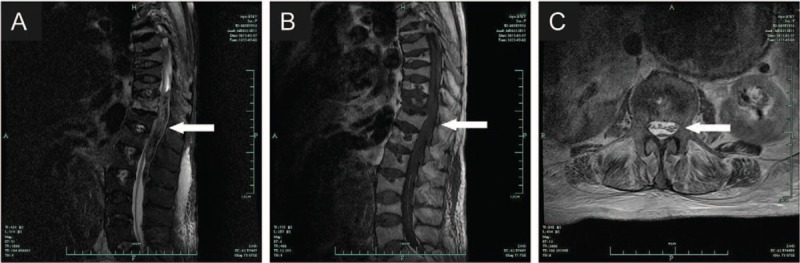
Magnetic resonance imaging performed 92 h after anesthesia and approximately 52 h after the onset of sensorimotor symptoms. Imaging revealed an epidural hematoma at the level of T11-L3 with compression of the spinal cord (arrows, panels A–C).

Stent graft implantation was scheduled by the interventional therapy group to treat her abdominal aortic aneurysm under single-dose spinal anesthesia. Spinal anesthesia was performed by an experienced anesthesiologist at the L3–L4 interspace using a 27-g Quincke needle (Portex, Kent, England). Puncture was successful on the first attempt and revealed clear cerebral spinal fluid in the hub of the needle. In response, 10 mg of hyperbaric ropivacaine was injected intrathecally. The procedure was uneventful; therefore, 30 mg of heparin was administered for intraoperative anticoagulation. At the end of surgery, the patient was able to move both legs and was transferred onto her hospital bed with assistance. Postoperative laboratory tests revealed that she had a significant low hemoglobin concentration (64 g/L) and a low platelet count (55 × 10^9^/L). Forty hours after surgery, the patient complained of numbness and swelling of her lower extremities. Later that same day, the patient reported progressive paralysis that was developing in both legs accompanied with sensory disturbances. Eight hours later, a physical examination revealed a 0/5 score on the Muscle Strength Grading System (MSGS). After unsuccessful medical treatment with mannitol, mecobalamin, and methylprednisolone for 2 days, in addition to fresh frozen plasma (410 mL) and packed red blood cells (2 units) infusion, emergent magnetic resonance imaging (MRI) detected an epidural hematoma at the level of T11-L3 with compression of the spinal cord (Fig. 2).

Eighty hours after the onset of sensorimotor symptoms, lumbar decompression and removal of the hematoma were performed via laminectomy at L2–L3 under general anesthesia. During surgical exploration, a tense epidural hematoma was found around L2. Postoperatively, the back pain resolved and paralysis of the lower limbs gradually recovered. The patient was discharged 2 months after the operation with a 3/5 score on the MSGS. Twelve months later, follow-up examination revealed that her muscle strength had recovered steadily and gradually to 4+/5 on the MSGS.

## 3 Discussion

Stent graft implantation is the conventional therapy for abdominal aortic aneurysms. Further, general anesthesia or intrathecal block is the routine anesthesia of choice at most institutions. In patients with compromised respiratory function, an intrathecal block might be the first choice.^[5]^ However, heparinization during the procedure and potential coagulation dysfunction resulting from a dissected aortic aneurysm may complicate or prevent the use of intrathecal puncture.^[6,7]^ Although reports suggest that the incidence of epidural hematoma is much lower following a subarachnoid block than an epidural block,^[8]^ epidural hematomas still occasionally occur. In the present case, the patient had normal preoperative coagulation function and platelet count, but poor to low hemoglobin levels and low platelet counts postoperatively, thereby suggesting the over-consumption of platelets due to the presence of a giant aortic aneurysm. Therefore, intrathecal block should be reconsidered as the anesthetic choice for those patients.

An epidural hematoma can have catastrophic complications. MRI is the best diagnostic technique in cases of suspected spinal hematoma; however, MRI scanners use strong magnetic fields to generate images of the organs in the body, which may place patients with metal implants in danger of stent displacement. In the present case, reluctance toward performing MRI examination within the first 2 days after surgery was largely due to fear of stent displacement due to the powerful magnetic field. The examination results confirmed that an intra-aortic stent is safe for MRI scanning.

Once the diagnosis of an epidural hematoma has been established, prompt neurosurgical intervention and decompression are crucial to achieve functional neurological recovery. The usual recommendation is laminectomy within 8 h of onset.^[9]^ However, there are rare cases in which delayed intervention can lead to partial or full recovery.^[10]^ In the present case, the patient underwent surgical intervention within 80 h and still achieved a nearly full recovery. To the best of our knowledge, this is the longest delay in which motor recovery was achievable, thereby suggesting that a larger window for therapy may exist in patients with epidural hematoma-induced paralysis.

## 4 Conclusions

Due to potential coagulation dysfunction, an intrathecal block should be avoided, if possible. In addition, MRI is feasible in detecting epidural hematoma, even in the presence of an intra-aortic stent. Decompression with laminectomy remains a viable treatment option, even if the diagnosis is established much later than 8 h after the onset of hematoma-induced symptoms.

## Acknowledgments

The authors thank Editage (www.editage.com) for English language editing.

## Author contributions

**Conceptualization:** Y.-X. Yao.

**Writing – original draft:** M.-X. Li.

**Writing – review & editing:** L.-J. Sun.
